# Contemporary AI attitude scale research: a systematic mapping review and bibliometric analysis

**DOI:** 10.3389/fpsyg.2026.1956889

**Published:** 2026-10-02

**Authors:** Yusuf Ziya Olpak

**Affiliations:** Department of Computer Education and Instructional Technology, Faculty of Education, Kirşehir Ahi Evran University, Kirşehir, Türkiye

**Keywords:** artificial intelligence, attitudes toward AI, bibliometric analysis, measurement instruments, systematic mapping review

## Abstract

**Introduction:**

The rapid expansion of artificial intelligence (AI) has been accompanied by growing efforts to measure individuals' attitudes toward these technologies. However, existing AI attitude scales vary considerably in their theoretical foundations, dimensional structures, target populations, and application contexts, raising questions about the extent to which they represent equivalent conceptualizations of the construct.

**Methods:**

This systematic mapping review examined contemporary AI attitude scale research and mapped its thematic, intellectual, methodological, and conceptual structure. A predefined Web of Science Core Collection search identified 298 eligible AI-related psychometric scale studies published between 2022 and June 2026; within this corpus, AI attitude was the most frequently represented construct, with 51 studies forming the final analytic sample. The review integrated descriptive and conceptual synthesis with bibliometric mapping analyses, including author keyword and abstract-term co-occurrence, co-citation, and bibliographic coupling.

**Results:**

The findings showed that scale development and psychometric evaluation constitute prominent methodological themes in the literature. Co-citation analysis linked the broader technology-acceptance tradition with AI-specific approaches to attitude measurement, while bibliographic coupling revealed four interconnected contemporary research fronts involving scale refinement and contextual extension, education and generative AI, population- and domain-specific measurement, and cross-cultural validation around established scale families. Despite these overlapping knowledge bases, substantial conceptual heterogeneity was evident across instruments, including unidimensional, positive-negative, cognitive-affective-behavioral, functional, ethical, and domain-specific multidimensional conceptualizations.

**Discussion:**

Overall, the findings indicate that bibliographic and methodological commonality has not produced conceptual uniformity in AI attitude measurement. Future research should therefore prioritize explicit construct definition, conceptual fit between instruments and research purposes, measurement invariance, cross-cultural validation, and cumulative evaluation of established measures rather than assuming equivalence across scales carrying the same construct label.

## Introduction

Artificial intelligence (AI) is becoming increasingly embedded in how individuals learn, work, communicate, obtain information, and make decisions. As encounters with AI become a routine feature of everyday and professional life (Sarun et al., 2026), understanding how individuals evaluate and respond to these technologies has become an important concern across disciplines. In this context, attitude represents a particularly relevant psychological construct because it captures individuals' evaluative orientations toward an object — such as a person, group, idea, behavior, product, or event. Eagly and Chaiken (1993), p. 1 defined attitude as “a psychological tendency that is expressed by evaluating a particular entity with some degree of favor or disfavor.” From this perspective, attitudes toward AI can be understood broadly as individuals' favorable or unfavorable evaluations of AI and its implications. Such evaluations are especially important because AI may simultaneously be perceived as beneficial and promising in terms of productivity, decision-making, scientific progress, and problem solving, while also generating concerns related to privacy, fairness, accountability, and human control (OECD, 2024; O'Shaughnessy et al., 2023). Importantly, favorable and unfavorable evaluations of AI are not necessarily mutually exclusive; individuals may recognize the benefits of AI while simultaneously expressing concerns about its risks and consequences (Schepman and Rodway, 2020; 2023).

The growing importance of AI attitudes has been accompanied by increasing efforts to measure them. However, attitudes toward AI have not been operationalized uniformly across existing instruments. A variety of scales have been developed to assess attitudes toward AI across different populations and contexts, with considerable variation in their dimensional structures. Some measures conceptualize AI attitude as a unidimensional construct representing an overall evaluative orientation toward AI (Grassini, 2023; Stein et al., 2024), whereas others distinguish positive and negative attitudes as separate dimensions (Schepman and Rodway, 2020, 2023, 2026). Still other instruments adopt more differentiated structures encompassing cognitive, affective, behavioral, ethical, functional, or context-specific components. This diversity is reflected in the markedly different dimensional structures reported across existing scales, ranging from unidimensional measures (Grassini, 2023; Novotny et al., 2026) to instruments comprising two (Cheng et al., 2025; Marengo et al., 2025), three (Suh and Ahn, 2022; Sultana et al., 2025), four (Aydin Yildiz et al., 2026; Durak et al., 2026), five (Ahmadi Fatalaki et al., 2025), or six dimensions (Park et al., 2024). Such variation indicates that differences among AI attitude measures concern not merely the number of dimensions identified, but more fundamentally what psychological responses are considered constitutive of an attitude toward AI.

This conceptual variation becomes particularly important when the substantive content of the dimensions is considered. Instruments with the same number of factors do not necessarily capture the same aspects of AI attitude, and dimensions bearing different labels may in fact overlap in evaluative content. For instance, the two-factor scale developed by Schepman and Rodway (2023) comprises “positive attitudes toward AI” and “negative attitudes toward AI,” whereas the two-factor scale validated by Svetlanova et al. (2026) comprises “operational and workforce impact” and “clinical benefits” — despite both instruments sharing the same two-factor structure. Similarly, substantial conceptual variation is evident among three-factor measures, with dimensions encompassing “cognitive,” “affective,” and “behavioral” components in Suh and Ahn (2022), “determination,” “exploration,” and “collaboration” in Sultana et al. (2025), and “risks and limitations,” “technical advantages,” and “personal advantages” in Bilge et al. (2025). Consequently, scales carrying the common label of “AI attitude” may not necessarily operationalize equivalent psychological content. This creates an important measurement problem: observed differences between studies may reflect genuine differences among populations or contexts, but they may also arise because ostensibly similar instruments measure different aspects of AI-related evaluation.

A further source of heterogeneity concerns the specificity of the attitude object. Some instruments are designed to capture general evaluations of AI without restricting the attitude object to a particular application or setting (Grassini, 2023; Schepman and Rodway, 2023, 2026), whereas a number of instruments assess attitudes within specific contexts, such as healthcare (Karaarslan and Ergin, 2026), defense (Hadlington et al., 2023), education (Ahmadi Fatalaki et al., 2025), engineering (Mirsch et al., 2026), and nursing (Yilmaz et al., 2025). Although context-specific measures can provide valuable information about individuals' evaluations of AI within particular domains, their dimensions may partly reflect characteristics and concerns unique to those domains. For example, the dimensions of “risks and limitations,” “technical advantages,” and “personal advantages” identified in the context of AI in psychotherapy reflect considerations directly related to that application context (Bilge et al., 2025). Thus, differences among AI attitude measures may arise not only from alternative theoretical conceptualizations of attitude but also from differences in the specificity of the attitude object and the context in which it is evaluated.

The conceptual heterogeneity observed across AI attitude measures also raises a broader theoretical question concerning what constitutes an attitude toward AI. One useful framework for addressing this question is the tripartite conceptualization of attitude, which distinguishes cognitive, affective, and behavioral forms of attitudinal response (Breckler, 1984). Within this framework, the cognitive component encompasses beliefs, thoughts, and evaluations concerning the attitude object, the affective component refers to emotional responses and feelings toward it, and the behavioral component involves action tendencies and behavioral intentions in relation to the object (Breckler, 1984; Suh and Ahn, 2022). Importantly, these components represent different forms of attitudinal response rather than different evaluative directions. Breckler (1984) proposed that all three components vary along a common evaluative continuum: affective responses may be pleasurable or unpleasurable, behavioral responses may be favorable or unfavorable, and cognitive responses may likewise involve favorable or unfavorable thoughts. This distinction is particularly relevant to AI, because individuals may simultaneously recognize potential benefits, experience positive emotions, and demonstrate willingness to engage with AI while also perceiving risks, experiencing concern, or showing reluctance toward particular forms of AI use. Accordingly, examining existing instruments requires attention not only to their number of dimensions, but also to the psychological content and evaluative direction represented by those dimensions.

The increasing number and diversity of AI attitude measures have therefore created a literature in which instrument proliferation does not necessarily translate into cumulative measurement development. When scales differ in their theoretical foundations, target objects, dimensional structures, and substantive content, findings obtained with different instruments cannot automatically be assumed to reflect equivalent conceptualizations of the construct. This limits direct comparison across studies, complicates the interpretation of apparently inconsistent findings, and makes it more difficult to determine which conceptualizations have become influential within the field. The issue is further complicated by the continuing development, adaptation, shortening, and cross-cultural validation of AI attitude scales. Although these developments expand the availability and international applicability of measurement instruments, they also increase the need to understand whether the field is converging around shared conceptual foundations or continuing to develop along distinct measurement traditions.

Several reviews have examined attitudes toward AI, although their objectives and scopes differ from the broader measurement-oriented focus of the present study. Amiri et al. (2024), for example, synthesized evidence concerning AI attitudes and knowledge among medical, dental, and nursing students, whereas Wu et al. (2025) reviewed responses, attitudes, and utilization behaviors related to generative AI in higher education. More recently, Bagočiunaite and Endriulaitiene (2026) specifically examined how employee attitudes toward AI have been defined and measured in workplace research, identifying substantial variation in measurement approaches and highlighting the resulting challenges for cross-study comparability. Although these reviews provide valuable insights into attitudes toward AI within particular populations, contexts, or applications, none of them provides a cross-context synthesis focused specifically on AI attitude measurement instruments while simultaneously examining the thematic and intellectual structure of the field. Consequently, there remains a need to understand how contemporary AI attitude scales conceptualize and operationalize the construct across different populations and application contexts, how their methodological characteristics vary, which intellectual foundations have shaped this measurement literature, and how different strands of AI attitude scale research are related within the broader research landscape.

Addressing this gap requires more than cataloging available instruments. A systematic mapping review can systematically identify, classify, and synthesize study- and instrument-level characteristics of the literature, including publication patterns, methodological practices, dimensional structures, and conceptualizations of AI attitude. However, such synthesis alone provides limited insight into the broader thematic and intellectual organization of the field. Bibliometric mapping analysis (BMA) provides a complementary perspective by revealing patterns that emerge from relationships among publications, terms, and cited sources. In particular, author keyword and abstract-term co-occurrence analyses can identify prominent thematic patterns and relationships; co-citation analysis can reveal the intellectual foundations on which contemporary AI attitude scale research draws; and bibliographic coupling can identify relationships among contemporary studies based on their shared knowledge bases. Integrating a systematic mapping review with BMA therefore enables AI attitude measurement research to be examined at two complementary levels: the substantive and methodological characteristics of individual studies and instruments, and the thematic and intellectual structure within which this measurement literature has developed.

Against this background, the present study makes three complementary contributions to the literature on AI attitude measurement. Conceptually, it synthesizes how AI attitude has been conceptualized and operationalized across contemporary measurement instruments, with particular attention to dimensional structure, evaluative direction, attitudinal components, and the distinction between general and context-specific attitude objects. Methodologically, it integrates a systematic mapping review with BMA to connect study- and instrument-level characteristics with the thematic and intellectual structure of AI attitude scale research. Practically, it identifies areas of conceptual convergence and divergence across existing instruments, providing evidence that can inform the selection and interpretation of AI attitude measures and guide the development, adaptation, and validation of future instruments. Accordingly, the present study examines the thematic and intellectual structure of contemporary AI attitude scale research, the publication and methodological characteristics of the included studies, and the ways in which AI attitude has been conceptualized and operationalized across existing instruments. Against this background, the study addressed the following research questions:

1. What thematic patterns characterize contemporary AI attitude scale research, as reflected in author keywords and abstract terms?
2. What intellectual foundations and contemporary research relationships characterize AI attitude scale research, as revealed by co-citation and bibliographic coupling analyses?
3. What publication and methodological patterns characterize studies involving the development, validation, adaptation, and psychometric evaluation of AI attitude scales?
4. How has AI attitude been conceptualized across existing scales, and what areas of conceptual convergence and divergence can be identified?

## Methods

This study integrates a systematic mapping review with BMA to provide complementary perspectives on contemporary research on AI attitude measurement. The systematic mapping review was designed to identify, classify, and descriptively synthesize the characteristics and conceptual structure of contemporary AI attitude scale research within a predefined bibliographic corpus, rather than to conduct a formal appraisal of the methodological quality of individual validation studies or the strength of evidence supporting specific measurement properties. The identification and study-selection process was reported with reference to the PRISMA 2020 guidelines (Page et al., 2021) to enhance transparency and completeness in reporting. Within this framework, AI-related psychometric scale studies were systematically identified, selected, and classified, after which the AI attitude studies were examined in greater depth with respect to their conceptual and methodological characteristics.

BMA complements the systematic mapping review by providing a quantitative perspective on the development and intellectual structure of the research field. BMA employs bibliographic and citation-based techniques, together with computer-assisted data processing, to examine patterns and relationships within a body of scientific literature (Wallin, 2005; Ellegaard and Wallin, 2015). By integrating statistical analysis with visualization techniques, it enables the identification of relationships, thematic patterns, and research trends within the literature. Furthermore, the use of computer algorithms and visualization tools allows BMA to objectively summarize large volumes of bibliographic data, providing reliable and reproducible insights into the characteristics and evolution of the research field (Aria and Cuccurullo, 2017; Martins et al., 2024).

Integrating the systematic mapping review and BMA was particularly appropriate for the objectives of the present study because neither approach alone provides a complete account of AI attitude scale research. BMA enables the identification of prominent themes through author keywords and abstract terms, as well as the intellectual foundations of the field through citation-based relationships, whereas the systematic mapping review allows for a structured examination and classification of the publication and methodological characteristics of the included studies and the ways in which AI attitude has been conceptualized across existing scales. Together, these complementary approaches provide a multidimensional perspective that combines field-level bibliometric patterns with study- and instrument-level evidence, thereby offering a more integrated understanding of the current state and conceptual structure of AI attitude scale research.

### Data collection

In review studies, the selection of the literature is typically guided by predefined criteria, such as publication source (Zawacki-Richter and Latchem, 2018), journal characteristics (Khorasanizadeh et al., 2024), indexing status (Olpak, 2022), or database coverage (Suárez et al., 2018). Accordingly, the present study adopted a deliberately defined bibliographic scope and restricted the search to articles published in journals indexed in the Science Citation Index Expanded (SCI-EXPANDED), Social Sciences Citation Index (SSCI), Arts and Humanities Citation Index (A&HCI), and Emerging Sources Citation Index (ESCI) within the Web of Science (WoS). The purpose of this restriction was to establish a consistent and clearly defined source base for the review rather than to imply that studies indexed outside WoS are necessarily of lower methodological quality. To maintain consistency throughout the screening and analysis process, and given the predominant role of English in international scientific communication (Chen, 2023), only articles published in English were considered. The review was further restricted to studies published from 2022 onward. This temporal boundary was deliberately selected to focus the review on the contemporary period of rapid expansion in AI technologies and AI-related research following the public release of ChatGPT by OpenAI in November 2022 (Lim et al., 2023). The release of ChatGPT represented an important turning point in the widespread public adoption and visibility of generative AI, reaching 100 million users within 2 months of its launch. Accordingly, the 2022–2026 period was used to examine the recent development of AI-related psychometric measurement during a period characterized by particularly rapid technological diffusion and growing research interest. The purpose of this temporal restriction was therefore not to provide an exhaustive historical account of all AI-related measurement instruments, but to establish a clearly defined contemporary scope for the review.

The literature search was conducted in WoS on June 24, 2026, using the following criteria: “Title = (‘artificial intelligence' or ‘AI' or ‘ai') AND Title = (‘scale' or ‘instrument'), Language = English, Document Type = Article, Early Access, or Review, Publication Years = 2022–2026, and Indexes = SCI-EXPANDED, SSCI, A&HCI, or ESCI.” The search was designed to identify contemporary studies in which AI-related measurement instruments constituted an explicit focus of the publication. Restricting the search terms to the title field provided a focused and reproducible strategy for constructing the review corpus. At the same time, this approach represents a deliberate boundary of the review and does not imply exhaustive retrieval of every AI-related psychometric instrument published during the study period. The initial search yielded 793 records. From this stage onward, all screening, eligibility assessment, and construct classification procedures involved two reviewers. Before the screening process began, the second reviewer was trained on the objectives of the review, the predefined inclusion and exclusion criteria, and the criteria used to identify and classify AI-related psychometric scales. Both reviewers screened the titles and abstracts of all retrieved records to determine whether they reported the development, validation, cross-cultural adaptation, or psychometric evaluation of an AI-related scale. Any differences in judgment at any stage were discussed with reference to the predefined criteria, and no record proceeded to the subsequent stage until consensus was reached between the two reviewers. During title and abstract screening, 482 records were excluded. Of these, 481 did not report the development, validation, cross-cultural adaptation, or psychometric evaluation of an AI-related scale (e.g., Hutzschenreuter et al., 2025; Roche et al., 2026; Tsang and Yin, 2026), and one was a correction notice for the study by Cipriani et al. (2026), which was already represented by the original article.

The remaining 311 articles were assessed in full text for eligibility by both reviewers. At this stage, 13 studies were excluded because they addressed scale-development methodology or employed other study designs without reporting the development, validation, cross-cultural adaptation, or psychometric evaluation of an AI-related scale (e.g., Beghetto et al., 2025; Rabkin, 2026). Survey protocols, *ad hoc* questionnaires, opinion polls, and studies using non-validated instruments were also excluded in accordance with the predefined eligibility criteria (see Table 1). Following full-text eligibility assessment, 298 studies met the broad eligibility criteria for AI-related psychometric scale research. These studies were subsequently classified by both reviewers according to the primary construct assessed by each instrument. Classification was based primarily on the construct explicitly identified by the instrument developers and, where necessary, was further informed by the stated purpose, conceptual definition, and dimensional structure of the instrument. All 298 eligible studies were classified according to their corresponding construct categories (see the Supplementary material). This classification showed that AI attitude was the most frequently represented construct within the eligible corpus (*n* = 51). Accordingly, the 51 studies classified as AI attitude studies constituted the focal analytic sample for the subsequent descriptive, conceptual, and bibliometric analyses. The overall process from study identification and eligibility assessment to construct classification and selection of the focal analytic sample is summarized in Figure 1.

**Table 1:** Eligibility criteria and selection of the focal AI attitude analytic sample.

Stage	Exclusion criteria	Inclusion criteria
Broad eligibility	Publications not indexed in SCI-EXPANDED, SSCI, A&HCI, or ESCI within the Web of Science Core Collection.	Articles published in journals indexed in SCI-EXPANDED, SSCI, A&HCI, or ESCI within the Web of Science Core Collection.
Broad eligibility	Publications not written in English.	Articles published in English.
Broad eligibility	Publications outside the 2022–2026 time period.	Articles published between 2022 and 2026.
Broad eligibility	Document types other than article, early access, or review.	Article, early access, or review publications.
Broad eligibility	Studies that did not report the development, validation, cross-cultural adaptation, or psychometric evaluation of an AI-related scale.	Studies reporting the development, validation, cross-cultural adaptation, or psychometric evaluation of standardized AI-related psychometric scales.
Focal analytic sample	Studies classified as assessing constructs other than AI attitude	Among the 298 eligible studies, those classified as assessing AI attitude were retained for the focal analytic sample

**Figure 1:**
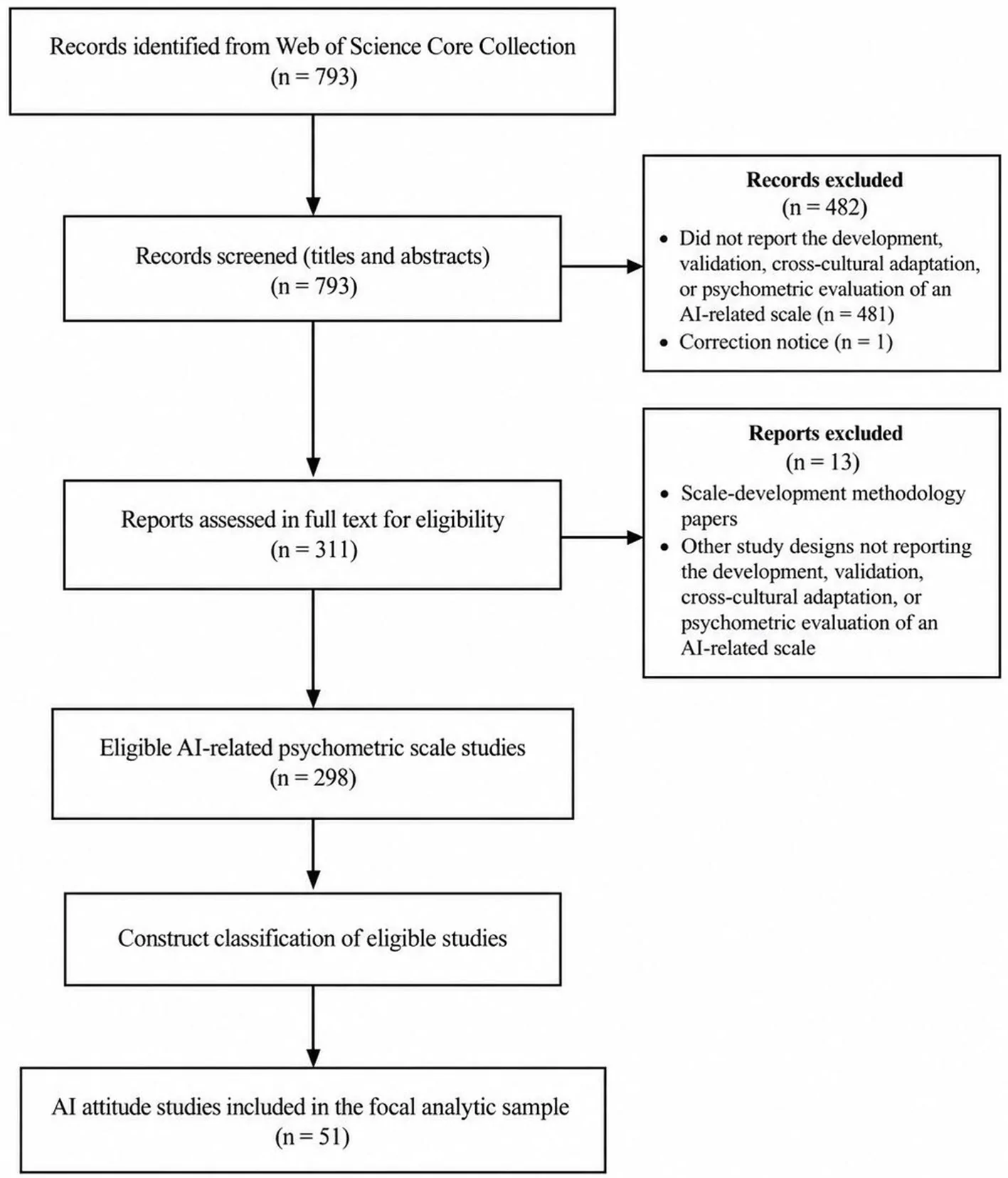
Flow diagram of the study selection process.

### Data analysis

The data were analyzed using performance analysis, descriptive synthesis, and BMA. Performance analysis was used to examine publication and authorship patterns in the reviewed literature. Frequencies and percentages were used as part of the descriptive synthesis to summarize study- and instrument-level characteristics, including journal indexing, sample countries, target populations, sample sizes, study types, response formats, scale dimensionality, and number of items. To systematically extract and organize these data, a publication classification form (PCF) was developed by adapting the classification forms used in previous research (e.g., Olpak, 2022; Olpak and Yilmaz, 2022). The PCF was also used to organize information relevant to the conceptual examination of AI attitude scales. In particular, the full texts of the included studies were examined to determine how AI attitude was conceptualized, the theoretical or conceptual foundations underlying the instruments, their dimensional structures, and the specific attitude components represented by their dimensions and items. These data were compared across studies to identify recurring conceptualizations as well as differences in how the construct of AI attitude was represented across general and context-specific instruments. This study-level examination complemented the bibliometric analyses by enabling patterns identified at the level of the research field to be considered alongside the conceptual characteristics of individual instruments.

BMA was conducted using VOSviewer (version 1.6.20), Centre for Science and Technology Studies (CWTS), Leiden University, Leiden, the Netherlands to examine the thematic and intellectual structure of AI attitude scale research. Prior to network construction, bibliographic data were checked for inconsistencies in terminology, spelling, abbreviations, singular and plural forms, and synonymous expressions. Where appropriate, semantically equivalent terms were standardized using a VOSviewer thesaurus file to reduce artificial fragmentation of the resulting networks. The minimum occurrence or citation thresholds applied in each analysis were determined with consideration of the size and distribution of the relevant dataset and are reported with the corresponding results to ensure transparency and reproducibility. Four complementary analyses were performed: (a) author keyword co-occurrence analysis, (b) abstract term co-occurrence analysis, (c) co-citation analysis of cited references, and (d) bibliographic coupling analysis of documents. The binary counting method was used for the author keyword and abstract term co-occurrence analyses. Co-citation analysis was performed to identify the intellectual foundations of the field, whereas bibliographic coupling analysis was conducted to examine relationships among the included studies based on their shared cited references. Taken together, the co-occurrence, co-citation, and bibliographic coupling analyses provided complementary perspectives on the thematic organization, intellectual foundations, and contemporary structure of the field.

## Results

### Performance analysis: publication year and authorship distribution

Table 2 presents the distribution of the reviewed studies by publication year and authors per article. Of the 51 studies included in the review, the largest proportion was published in 2025 (*n* = 25, 49.02%), followed by 2026 (*n* = 17, 33.33%). Given that the literature search was conducted in June 2026, the publication count for 2026 reflects only studies indexed up to the search date. Nevertheless, 17 studies had already been published by mid-2026, indicating strong publication momentum in this field. Regarding authorship, studies with three authors were the most common (*n* = 14, 27.45%), followed by those with two authors (*n* = 13, 25.49%) and four authors (*n* = 11, 21.57%). Single-author studies were relatively uncommon (*n* = 2, 3.92%), indicating that most research on AI attitude scales is conducted collaboratively.

**Table 2:** Distribution of the included studies by publication year and authors per article.

Publication year	Authors per article									Total
	1	2	3	4	5	6	7	8	10	
2022		1	1							**2**
2023	1	1			1					**3**
2024			3	1						**4**
2025		5	8	8	1		2	1		**25**
2026	1	6	2	2	2	3			1	**17**
**Total**	**2**	**13**	**14**	**11**	**4**	**3**	**2**	**1**	**1**	**51**

Bold values indicate row and column totals.

### Author keyword co-occurrence analysis

Among the 51 studies included in the review, one study (Schepman and Rodway, 2023) did not report author keywords. Consequently, this study was excluded from the keyword co-occurrence analysis, which was performed using the author keywords from the remaining 50 studies. Before conducting the analysis, author keywords were standardized to reduce terminological variation. Specifically, “attitudes” was standardized as “attitude” while “AI” was replaced with “artificial intelligence.” In addition, “artificial intelligence attitude,” “AI attitude,” “attitude toward AI,” “attitude toward artificial intelligence,” and “attitudes toward AI” were standardized as “attitudes toward artificial intelligence.”

Figure 2 illustrates the keyword co-occurrence network of the included studies. Using a minimum occurrence threshold of five, seven keywords were included in the network and grouped into two clusters. Artificial intelligence was the most frequently occurring and most strongly connected keyword (occurrences = 34, total link strength (TLS) = 34), followed by attitude (17, 24), scale development (13, 17), attitudes toward artificial intelligence (11, 11), reliability (5, 10), validity (5, 10), and generative artificial intelligence (5, 6). The first cluster reflects the conceptual and developmental orientation of the literature, linking AI-related attitude terminology with scale development and the emerging focus on generative AI. The second cluster reflects the psychometric evaluation of these instruments, characterized by the close association between reliability and validity. Taken together, the network indicates two closely related strands of AI attitude scale research: the conceptualization and development of measurement instruments and the evaluation of their psychometric properties.

**Figure 2:**
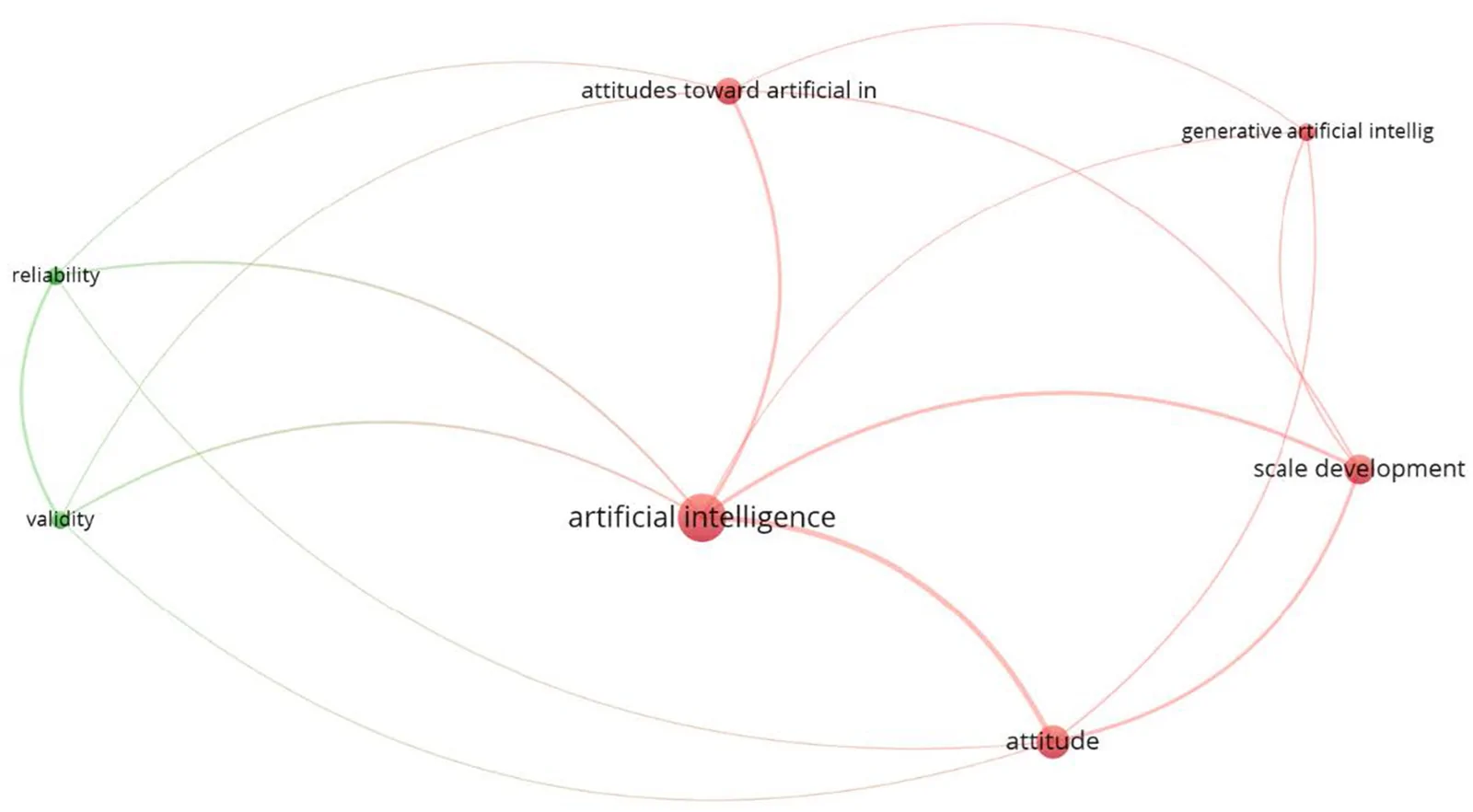
Author keyword co-occurrence network.

### Abstract term co-occurrence analysis

Figure 3 presents the term co-occurrence network generated from the abstracts of the included studies. Before the analysis, a thesaurus file was applied to exclude generic or analytically uninformative terms. Using binary counting, a minimum occurrence threshold of 10 was applied. Of the 1,467 terms identified in the abstracts, 19 met this threshold. Following VOSviewer's default relevance-based selection procedure, the 60% most relevant terms were retained, resulting in 11 terms being included in the final network. All 11 terms formed a single interconnected cluster. The most frequently occurring terms were attitude (49 occurrences), scale (48), artificial intelligence (45), confirmatory factor analysis (42), validity (40), and reliability (32), followed by item (27), research (26), exploratory factor analysis (23), factor structure (21), and participant (18). The network was characterized by the co-occurrence of substantive terms related to AI attitudes with terms representing scale development and psychometric evaluation. In particular, the prominent presence of confirmatory factor analysis, exploratory factor analysis, factor structure, validity, and reliability indicates that factor-analytic procedures and the evaluation of measurement properties constitute a substantial component of the abstract-level discourse in contemporary AI attitude scale research. The formation of a single interconnected cluster further suggests that the conceptual focus on AI attitudes and the methodological focus on scale development and psychometric evaluation are closely integrated within this literature.

**Figure 3:**
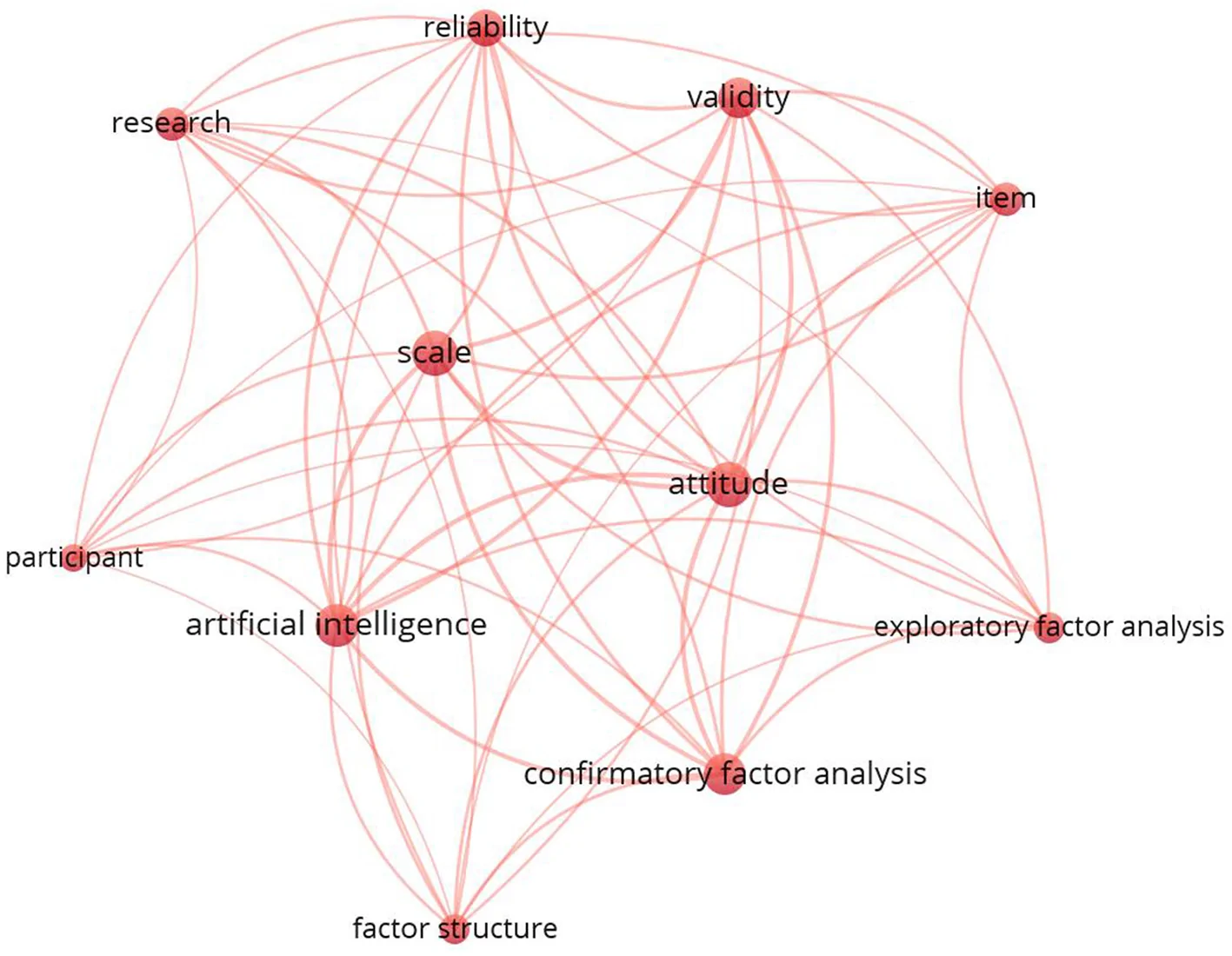
Abstract term co-occurrence network.

### Co-citation analysis

Figure 4 presents the co-citation network of cited references. Co-citation analysis was performed using cited references as the unit of analysis and the full counting method. Among the 2,392 cited references identified across the included studies, five met the minimum citation threshold of 17 and were included in the network analysis. These references formed a single interconnected cluster. Schepman and Rodway (2020) was the most frequently co-cited and most strongly connected reference in the network (citations = 36, TLS = 78), followed by Sindermann et al. (2021) (28, 65), Grassini (2023) (25, 59), Kaya et al. (2024) (19, 48), and Davis (1989) (19, 46).

**Figure 4:**
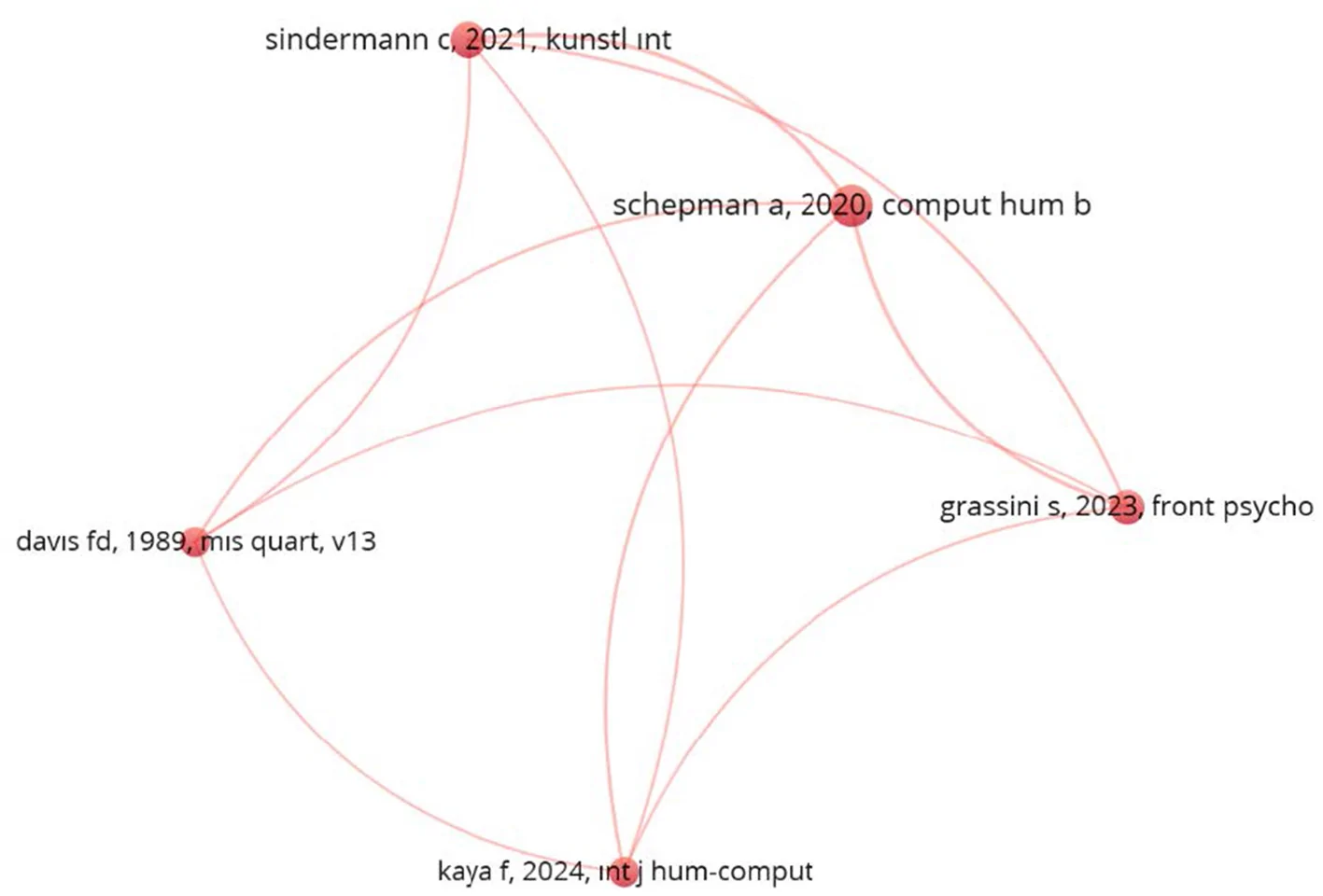
Co-citation network of cited references.

The composition of the co-citation network indicates that the intellectual foundations of contemporary AI attitude scale research encompass both the established technology-acceptance tradition and AI-specific approaches to attitude measurement. Davis (1989) represents the technology-acceptance tradition through the Technology Acceptance Model, whereas Schepman and Rodway (2020) conceptualized general attitudes toward AI in terms of positive and negative evaluations, including perceived benefits, concerns, and emotional responses. Sindermann et al. (2021) provided another AI-specific operationalization by conceptualizing attitudes in terms of acceptance and fear and examining this structure across German, Chinese, and UK samples. Grassini (2023) further contributed to general AI attitude measurement through the development of a brief unidimensional instrument, while Kaya et al. (2024) extended the general attitudes toward AI tradition through cross-cultural adaptation and the examination of AI attitudes in relation to AI anxiety, personality traits, and demographic characteristics. Overall, the co-citation of these references points to an intellectual foundation that connects the broader technology-acceptance tradition with increasingly AI-specific approaches to conceptualizing and measuring attitudes.

### Bibliographic coupling analysis of documents

Figure 5 presents the bibliographic coupling network of the included studies. Bibliographic coupling analysis was conducted using documents as the unit of analysis and the full counting method. No minimum citation threshold was imposed, allowing all 51 studies in the analytic corpus to be included in the analysis. The resulting network comprised four clusters, containing 16, 13, 12, and 10 studies, respectively, and showed extensive bibliographic connections both within and across clusters. The highest total link strength was observed for Satici et al. (2025) (TLS = 243), followed by Arees et al. (2025) (242), Köse et al. (2025) (233), Rózsa et al. (2025) (219), Novotny et al. (2026) (219), Boduroglu and Yigiter (2026) (218), and Grassini (2023) (215). Higher total link strength indicates greater overlap between a study's cited references and those of other documents in the network. Overall, the dense pattern of within- and cross-cluster links indicates that contemporary AI attitude scale studies draw on overlapping knowledge bases while forming several distinguishable bibliographic groupings.

**Figure 5:**
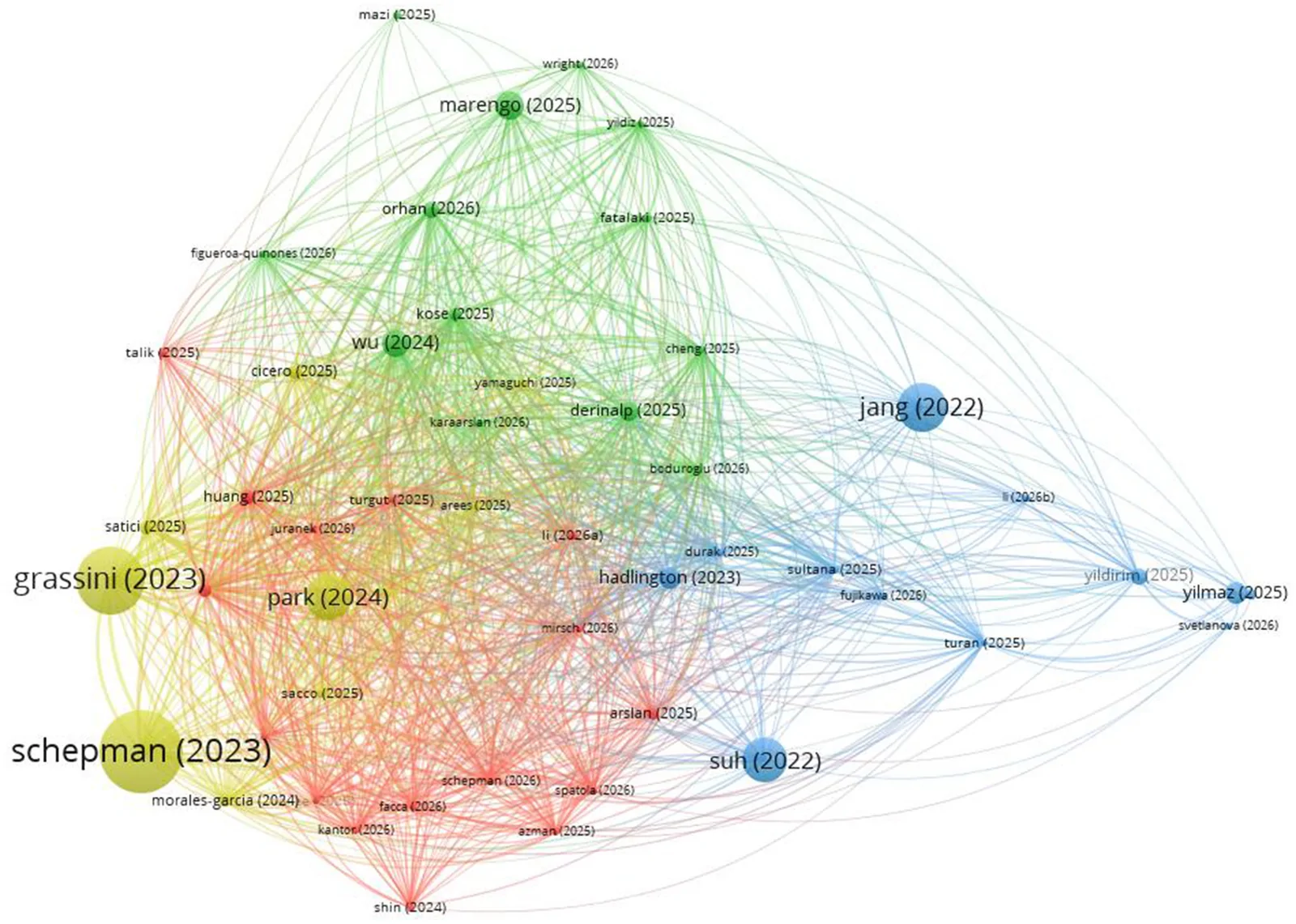
Bibliographic coupling network of ai attitude scale studies.

Substantive examination of the studies within the four bibliographic coupling clusters revealed partially distinguishable contemporary research fronts. Cluster 1 was characterized by the diversification, refinement, and contextual extension of AI attitude measurement. It combined adaptations and refinements of established measures with the development of newer general and context-specific instruments addressing settings such as psychotherapy, defense, and medicine. Cluster 2 was predominantly associated with education- and generative-AI-oriented scale development and validation, bringing together studies focusing on university students, EFL learners and teachers, generative AI, and AI-assisted learning, although several adjacent validation studies were also represented. Cluster 3 primarily reflected population- and domain-specific multidimensional measurement, with a notable concentration of instruments developed or validated for nursing, healthcare, defense, and student populations. Cluster 4 showed the clearest instrument-family structure, being dominated by studies associated with the General Attitudes toward Artificial Intelligence Scale (GAAIS) and the AI Attitude Scale (AIAS-4) traditions and their subsequent cross-cultural adaptations and validations. Taken together, the four clusters suggest that contemporary AI attitude scale research is developing along several interconnected trajectories: the continued refinement and contextual extension of AI attitude measurement, the rapid growth of education- and generative-AI-specific instruments, increasing development of population- and domain-sensitive multidimensional measures, and cumulative cross-cultural validation around established general-scale families. Importantly, the extensive links both within and across clusters indicate that these research fronts are not bibliographically isolated. Rather, they draw on overlapping cited knowledge bases while extending AI attitude measurement in different conceptual, cultural, and application-specific directions.

### Publication and methodological characteristics of AI attitude scale studies

Table 3 presents the publication and methodological characteristics of the AI attitude scale studies included in the review.

**Table 3:** Publication and methodological characteristics of AI attitude scale studies.

#	*f*	%
WoS index		
SSCI	24	47.06
SCI-EXPANDED/SSCI	14	27.45
ESCI	10	19.61
SCI-EXPANDED	3	5.88
Sample country		
Türkiye	16	31.37
China	5	9.80
United Kingdom	4	7.84
Multinational^*^	4	7.84
Korea	3	5.88
USA	3	5.88
Italy	2	3.92
Japan	2	3.92
Peru	2	3.92
France	1	1.96
Germany	1	1.96
Hungary	1	1.96
Iran	1	1.96
Kazakhstan	1	1.96
Malaysia	1	1.96
Poland	1	1.96
Qatar	1	1.96
Spain	1	1.96
Taiwan	1	1.96
Target population		
Adults/General population	24	47.06
Higher education students	9	17.65
Healthcare professionals/trainees	7	13.73
School students	5	9.80
Education professionals	4	7.84
Mixed participant groups^**^	2	3.92
Sample size		
≤ 250	5	9.80
251–500	11	21.57
501–750	17	33.33
751–1,000	11	21.57
1,001–1,500	2	3.92
≥1,501	5	9.80
Study type		
Original scale development	27	52.94
Adaptation/Psychometric validation	24	47.06
Response format		
4-point Likert	1	1.96
5-point Likert	40	78.43
7-point Likert	2	3.92
10-point Likert	7	13.73
Not reported	1	1.96
Scale dimensionality		
1	9	17.65
2	22	43.14
3	8	15.69
4	6	11.76
5	3	5.88
6	3	5.88
Number of items		
4	7	13.73
6	1	1.96
9	1	1.96
10	1	1.96
11	1	1.96
12	5	9.80
13	1	1.96
14	1	1.96
15	8	15.69
16	1	1.96
17	3	5.88
19	1	1.96
20	6	11.76
23	2	3.92
24	2	3.92
25	3	5.88
26	3	5.88
28	1	1.96
32	1	1.96
33	1	1.96
44	1	1.96

^*^Multinational studies include studies conducted simultaneously in more than one country (e.g., United Kingdom–USA; Germany–USA; Poland–USA–United Kingdom).

^**^Mixed participant groups refer to studies that included participants from more than one distinct population (e.g., engineering students and professionals or high school and university students) rather than focusing on a single target population.

### Journal indexing

Table 3 shows that most of the reviewed studies were published in journals indexed in SSCI, either exclusively (*n* = 24, 47.06%) or jointly with SCI-EXPANDED (*n* = 14, 27.45%). The remaining studies were published in ESCI-indexed journals (*n* = 10, 19.61%) or journals indexed exclusively in SCI-EXPANDED (*n* = 3, 5.88%). Overall, 38 of the 51 studies (74.51%) were published in journals with SSCI coverage.

### Sample countries, target populations and sample characteristics

As shown in Table 3, regarding the countries in which the study samples were recruited, Türkiye accounted for the largest proportion of the reviewed studies (*n* = 16, 31.37%), followed by China (*n* = 5, 9.80%), the United Kingdom (*n* = 4, 7.84%), and Korea and the United States (*n* = 3 each, 5.88%), while most other countries were represented by only a small number of studies. Multinational studies were also relatively uncommon (*n* = 4, 7.84%). Overall, these findings indicate that AI attitude scales have been examined across diverse geographical settings, although the evidence remains concentrated in a limited number of countries, particularly Türkiye.

With respect to participant characteristics, almost half of the reviewed studies targeted adults or the general population (*n* = 24, 47.06%), making this the most frequently investigated participant group. Higher education students constituted the second largest category (*n* = 9, 17.65%), followed by healthcare professionals and trainees (*n* = 7, 13.73%), with most studies in this category focusing on nursing-related populations, such as nurses, undergraduate nursing students, and primary healthcare nurses. Comparatively fewer studies focused on school students (*n* = 5, 9.80%), with study samples including, for example, children, K−12 students, and high school students, followed by education professionals (*n* = 4, 7.84%), whereas only two studies (3.92%) included mixed participant groups. These findings indicate that AI attitude scale research has primarily concentrated on adult populations, with more limited representation of school students, education professionals, and professionals outside the healthcare sector.

Sample sizes varied considerably across the reviewed studies, ranging from 116 to 4,092 participants (*M* = 825.90, SD = 766.73; Median = 678). Most studies included between 251 and 1,000 participants. The higher mean relative to the median reflects the influence of a small number of studies with particularly large samples, whereas the majority of studies were based on more moderate sample sizes.

### Scale development approaches

As shown in Table 3, the reviewed studies were almost evenly divided between original scale development (*n* = 27, 52.94%) and the adaptation or psychometric validation of existing instruments (*n* = 24, 47.06%). This pattern indicates that contemporary AI attitude measurement research encompasses both continued instrument development and substantial efforts to adapt and validate established measures across different populations and contexts. Among the existing instruments, the scales developed by Schepman and Rodway (2020, 2023) were the most frequently adapted (*n* = 7), followed by Grassini's (2023) AIAS-4 (*n* = 6) and the Student Attitudes Toward Artificial Intelligence Scale (SATAI) developed by Suh and Ahn (2022) (*n* = 3), indicating their prominent role in subsequent AI attitude measurement research.

### Response formats and scale characteristics

As shown in Table 3, the 5-point Likert format was clearly predominant, being used in 40 of the 51 studies (78.43%). Other response formats were considerably less common, with 10-point, 7-point, and 4-point scales used in a smaller number of studies. Overall, the findings indicate a strong preference for the 5-point Likert format in contemporary AI attitude scale research.

Considerable variation was observed in scale dimensionality. Two-dimensional structures were the most common (*n* = 22, 43.14%), followed by unidimensional (*n* = 9, 17.65%) and three-dimensional instruments (*n* = 8, 15.69%), while four-, five-, and six-dimensional structures were less frequent. Thus, although bidimensional conceptualizations were the most prevalent, no single dimensional structure characterized the literature as a whole.

Scale length also varied substantially, ranging from four to 44 items. Fifteen-item scales were the most common (*n* = 8, 15.69%), followed by 4-item (*n* = 7, 13.73%) and 20-item instruments (*n* = 6, 11.76%). Overall, the wide variation in item counts reflects considerable heterogeneity in the length of AI attitude measures.

### Conceptualization of AI attitude

The reviewed studies revealed considerable variation in how AI attitude was conceptualized. At the broadest level, the identified instruments could be classified into two broad categories: those assessing general attitudes toward AI (e.g., Yamaguchi et al., 2025) and those measuring attitudes toward AI within a specific population, technology, or application context (e.g., Öztürk Yildirim and Karaman, 2025). General instruments conceptualized AI as a broad technological entity and assessed individuals' overall evaluations of AI, whereas domain-specific instruments focused on attitudes toward specific AI applications within contexts such as nursing, psychotherapy, defense, engineering, and university admissions. Consequently, the object of evaluation varied considerably across the reviewed instruments, ranging from AI as a general technology to highly context-dependent AI applications.

One prominent conceptual approach treated AI attitude as a general evaluative disposition toward AI. Instruments adopting this perspective viewed attitude as an overall tendency to evaluate AI favorably or unfavorably without distinguishing separate underlying components. The AIAS-4 (Grassini, 2023) provided a clear example of this approach. Its subsequent linguistic adaptations and cross-cultural validation studies consistently retained the same unidimensional conceptualization across diverse linguistic and cultural contexts (Arslan et al., 2025; Juranek and Pochwatko, 2026; Köse et al., 2025; Morales-García et al., 2024; Satici et al., 2025; Talik et al., 2025). Similarly, the “General AI Attitude Scale (GAIA-15)” (Hering et al., 2026) and the “General AI Attitude Short Scale” (Novotny et al., 2026) conceptualized AI attitude as a unidimensional construct, with items representing all three facets of attitude—cognitive, affective, and behavioral.

Another conceptual approach viewed AI attitude as comprising distinct positive and negative evaluations rather than as a single general disposition. Within this perspective, favorable and unfavorable evaluations of AI were treated as related but independent dimensions, acknowledging that individuals may simultaneously recognize both the potential benefits and the possible risks of AI technologies. Positive attitudes generally reflected perceived usefulness, innovation, and expected benefits associated with AI, whereas negative attitudes captured concerns regarding ethical issues, loss of human control, privacy, job displacement, or other perceived risks. This conceptualization was first introduced in the GAAIS (Schepman and Rodway, 2020, 2023) and was subsequently retained in later developments and cross-cultural validation studies, including the “Short GAAIS-10” (Schepman and Rodway, 2026) and its Bahasa Malaysia (Azman et al., 2025), Chinese (Huang et al., 2025), Hungarian (Rózsa et al., 2025), Japanese (Yamaguchi et al., 2025), and Italian (Cicero et al., 2025; Sacco et al., 2025) versions. Similar positive–negative distinctions were also retained in several domain-specific instruments developed or adapted for defense (Facca and Béna, 2026; Hadlington et al., 2023), nursing (Hu et al., 2025; Yilmaz et al., 2025), and generative AI in higher education (Cheng et al., 2025; Marengo et al., 2025). Collectively, these findings indicate that many researchers conceptualized AI attitude as comprising distinct positive and negative evaluations, allowing favorable and unfavorable perceptions of AI to coexist within the same individual.

Among the instruments adopting a three-dimensional structure, however, the meaning assigned to these dimensions varied considerably. The SATAI (Suh and Ahn, 2022) was explicitly grounded in the classical tripartite model of attitude, distinguishing cognitive, affective, and behavioral components. More specifically, the cognitive component reflected beliefs and evaluations concerning the importance and necessity of AI education, the affective component captured positive evaluations of AI's personal and societal value, and the behavioral component represented intentions and willingness to engage with AI through continued learning, participation in AI-related activities, and future career aspirations. This conceptualization was retained in subsequent Turkish adaptation studies involving both emerging adults and secondary and high school students (Derinalp and Ozyurt, 2025; Turgut and Kunuroglu, 2025). Other three-dimensional instruments adopted substantially different conceptualizations. Rather than distinguishing cognitive, affective, and behavioral components, these scales defined attitude according to the particular aspects of AI being evaluated within a specific context. For example, the “Functional Attitudes toward Artificial Intelligence (FAAI)” scale (Li and Wang, 2026) was grounded in functional attitude theory and distinguished utility–knowledge, value-expression, and ego-defense functions, whereas the “Responsible AI Attitudes Specific to Engineering (RAISE)” scale (Mirsch et al., 2026) conceptualized attitudes toward responsible AI around the ethical principles of do-no-harm, transparency, and privacy. Similarly, the “Artificial Intelligence in Psychotherapy Scale (AIPS)” (Bilge et al., 2025) conceptualized attitudes through perceived risks and limitations, technical advantages, and personal advantages. These findings indicate that, although several AI attitude instruments employed a three-dimensional structure, there was little conceptual consensus regarding what the three dimensions should represent. Instead, the dimensions were defined according to the theoretical framework, application domain, and intended purpose of each instrument.

Beyond one-, two-, and three-dimensional conceptualizations, several instruments adopted more complex multidimensional structures, reflecting the increasing diversification of AI attitude research across application domains. Rather than organizing attitudes around a small number of broad evaluative dimensions, these instruments differentiated multiple aspects of AI that were specific to particular AI technologies and domains (e.g., generative AI and responsible AI), target populations (e.g., teachers and nurses), or application contexts (e.g., healthcare and psychotherapy). For example, the “EFL Teachers' Attitudes toward AI Scale” (Ahmadi Fatalaki et al., 2025) conceptualized attitudes through five dimensions—interest, ethical concerns, intention to use, usefulness for teachers, and usefulness for learners—reflecting both pedagogical and ethical evaluations of AI in language education. Similarly, the “Generative Artificial Intelligence Attitude Scale for EFL Learners (GenAIAS)” (Orhan et al., 2026) assessed university students' attitudes toward generative AI through the dimensions of learning/utility, enjoyment, usefulness, and interest, emphasizing learners' educational experiences and perceptions of generative AI in language learning. Comparable patterns were observed in healthcare-related instruments. The “Artificial Intelligence Attitude Scale for Nurses” (Öztürk Yildirim and Karaman, 2025) conceptualized attitudes through four dimensions—nursing care, organization, ethics, and AI readiness—highlighting the integration of professional responsibilities and preparedness for AI-assisted nursing practice. Similar multidimensional conceptualizations were also reported in other healthcare settings, including nursing education (Turan and Cengiz, 2025) and medicine (Kantor et al., 2026). Comparable multidimensional conceptualizations also emerged in occupational settings. For instance, to assess attitudes toward AI in the workplace, Park et al. (2024) developed a six-dimensional scale comprising perceived humanlikeness, perceived adaptability, perceived quality, AI-use anxiety, job insecurity, and personal utility, reflecting both favorable evaluations of AI and concerns about its potential consequences in the workplace. Collectively, these findings demonstrate that multidimensional AI attitude instruments increasingly define attitude in relation to the demands and characteristics of particular domains rather than relying on a common set of generic evaluative dimensions. Consequently, contemporary AI attitude research conceptualizes the construct in ways that are closely aligned with the technological, professional, and contextual realities in which AI is developed and used.

When considered alongside the bibliographic coupling results, the conceptual synthesis further indicates that bibliographic proximity does not imply conceptual equivalence. Cluster 4, for example, was dominated by studies associated with the GAAIS and AIAS-4 traditions and their cross-cultural adaptations, although these two scale families represent different dimensional conceptualizations of AI attitude. Cluster 2 was predominantly associated with education- and generative-AI-oriented instruments, whereas Cluster 3 showed a notable concentration of population- and domain-specific multidimensional measures. Cluster 1 reflected the diversification, refinement, and contextual extension of AI attitude measurement. Taken together, these findings indicate that contemporary AI attitude scale research draws on overlapping cited knowledge bases while continuing to exhibit substantial conceptual heterogeneity in how the construct is conceptualized.

## Discussion

The present study integrates a systematic mapping review with BMA to examine the contemporary landscape of AI attitude measurement within a predefined Web of Science–derived corpus covering the period from 2022 to June 2026. The findings reveal an important tension in this literature. On the one hand, contemporary studies draw on overlapping bibliographic knowledge bases and show recurring methodological emphases related to scale development and psychometric evaluation. On the other hand, substantial variation remains in how AI attitude itself is conceptualized. The central issue emerging from the review is therefore not simply the proliferation of instruments, but the coexistence of bibliographic interconnectedness with considerable conceptual heterogeneity. This distinction has important implications for how AI attitude scales are interpreted, compared, and selected for future research.

The concentration of publications in 2025 and the first half of 2026 indicates substantial recent research activity in AI attitude measurement. Although the present review was not designed to identify the causes of this increase, the pattern is consistent with the expanding presence of AI across educational, professional, healthcare, and everyday settings. More informative than publication growth alone is the nearly balanced distribution between original scale-development studies and studies involving adaptation or psychometric validation. Contemporary research therefore appears to involve two parallel processes: continued development of new instruments and continued examination of established measures across different populations and contexts. The repeated adaptation of instruments developed by Schepman and Rodway (2020, 2023), Grassini (2023), and Suh and Ahn (2022) further indicates that certain scale families have become recurring reference points in the contemporary measurement literature. Importantly, however, adaptation across languages or populations should not itself be interpreted as evidence of measurement equivalence; direct cross-cultural comparison ultimately requires explicit evidence that the measurement structure operates comparably across groups.

The bibliometric findings provide additional insight into the intellectual development of this emerging field. The co-occurrence analyses further clarify the methodological orientation of the field. The author keyword and abstract term analyses consistently showed that the most central and frequently occurring concepts were those related to scale development and psychometric evaluation, particularly artificial intelligence, attitude, scale development, reliability, and validity. These patterns indicate that instrument construction and psychometric evaluation constitute a prominent part of the discourse surrounding contemporary AI attitude research. Importantly, however, the frequent appearance of reliability, validity, or factor-analytic terminology should not be equated with evidence that all instruments have received equally comprehensive or rigorous psychometric evaluation. The present review mapped methodological characteristics but did not formally rate the quality of evidence for individual measurement properties. Accordingly, the findings identify what types of psychometric procedures are prominent in the literature, but they do not establish which existing instrument currently has the strongest overall evidence base. This distinction is important for interpreting apparent methodological convergence in the field.

The co-citation analysis provides a complementary perspective by revealing an intellectual foundation that connects the broader technology-acceptance tradition with AI-specific approaches to attitude measurement. The presence of Davis (1989) alongside Schepman and Rodway (2020), Sindermann et al. (2021), Grassini (2023), and Kaya et al. (2024) suggests that contemporary AI attitude measurement has developed at the intersection of established research on human responses to technology and more recent attempts to operationalize evaluations specifically directed toward AI. This relationship is theoretically important because technology acceptance and attitude toward AI should not automatically be treated as equivalent constructs. The conceptual synthesis showed that AI attitude measures extend beyond willingness to accept or use a technology and may include positive and negative evaluations, affective responses, perceived risks and benefits, ethical concerns, or behavioral tendencies. Consequently, future measurement research should make explicit how AI attitude is theoretically related to, but distinguished from, neighboring constructs such as acceptance, behavioral intention, anxiety, trust, readiness, and self-efficacy.

The bibliographic coupling analysis provides further evidence that contemporary AI attitude measurement does not constitute a single homogeneous research trajectory. Four partially distinguishable research fronts were identified. Cluster 1 reflected the diversification, refinement, and contextual extension of AI attitude measurement; Cluster 2 was predominantly associated with education- and generative-AI-oriented instruments; Cluster 3 contained a notable concentration of population- and domain-specific multidimensional measures; and Cluster 4 was dominated by the GAAIS and AIAS-4 traditions and their cross-cultural adaptations. Importantly, these clusters were extensively connected rather than bibliographically isolated. At the same time, their conceptual characteristics demonstrate that bibliographic proximity does not imply conceptual equivalence. Cluster 4 provides a particularly clear example: studies associated with the unidimensional AIAS-4 and the bidimensional GAAIS traditions share substantial bibliographic proximity even though the two scale families operationalize AI attitude differently. The network structure therefore points to overlapping cited knowledge bases rather than convergence around a single definition or dimensional model of AI attitude.

This distinction between bibliographic relatedness and conceptual equivalence is reinforced by the broader conceptual synthesis. Existing instruments represented several substantially different approaches to the construct, including a unidimensional general evaluative disposition, separate positive and negative evaluations, cognitive–affective–behavioral components, functional attitudes, ethical orientations, and multidimensional domain-specific conceptualizations. These approaches differ at a level more fundamental than the number of factors identified. For example, distinguishing positive and negative attitudes concerns evaluative direction, whereas distinguishing cognitive, affective, and behavioral components concerns different forms of attitudinal response. Treating these structures as interchangeable simply because both are multidimensional would obscure an important theoretical distinction. Similarly, instruments with three dimensions were shown to represent markedly different psychological content depending on whether they were grounded in the tripartite model, functional attitude theory, ethical principles, or the characteristics of a specific application domain. Thus, dimensionality alone provides limited information about what an AI attitude scale actually measures.

The increasing contextual specificity of many instruments raises an additional construct-boundary issue. Measures developed for education, healthcare, nursing, engineering, or psychotherapy often incorporate dimensions reflecting concerns that are particularly salient within those settings. Such contextualization may enhance the relevance of a measure for its intended application. At the same time, some instruments include dimensions involving constructs such as intention to use, readiness, anxiety, job insecurity, usefulness, or ethical concerns. Their inclusion raises a theoretically important question concerning whether these elements should be regarded as constitutive components of AI attitude itself or as related beliefs, antecedents, consequences, or contextual evaluations. This does not imply that such dimensions are inappropriate; rather, it emphasizes the need for instrument developers to define the attitude object and construct boundaries before determining the dimensional structure of a scale.

The methodological characteristics of the reviewed studies also provide valuable insights into the current stage of AI attitude scale research. The geographical distribution of the study samples provides additional insight into the current empirical landscape of the field. Although the reviewed studies included participants from a variety of countries, the evidence base was concentrated within a relatively small number of national contexts, with Türkiye contributing the largest proportion of study samples. Similarly, the distribution of target populations provides important insight into the current focus of AI attitude scale research. The predominance of studies targeting adults or the general population suggests that researchers have primarily focused on developing broadly applicable measures of AI attitudes rather than instruments intended for specific professional or demographic groups. The main implication is not that evidence obtained from these samples is inadequate, but that the generalizability and cross-cultural comparability of existing instruments should not be assumed. As the number of translations and cultural adaptations increases, measurement invariance studies across languages, countries, age groups, and professional populations would provide particularly valuable evidence concerning whether apparently equivalent scores represent the same underlying construct across groups.

One particularly noteworthy finding is the relatively balanced distribution between original scale-development studies and adaptation or validation studies. Approximately half of the reviewed publications focused on developing new instruments, whereas the remaining studies sought to adapt, translate, or validate existing measures. This balance suggests that AI attitude scale research is not driven solely by the development of new instruments. Instead, increasing attention is being devoted to examining the applicability of established measures across different languages, cultures, populations, and application contexts. Such a pattern is consistent with a research area that is gradually moving beyond initial instrument proliferation toward the refinement and broader evaluation of existing measures. This transition is also reflected in the methodological characteristics of the reviewed scales. While researchers appear to have converged on certain methodological practices, considerable variation remains in the structural characteristics of AI attitude instruments. The overwhelming preference for the 5-point Likert response format suggests the emergence of a common approach to response scaling. This preference is unsurprising, as 5-point Likert scales are widely regarded as providing an effective balance between measurement sensitivity and respondent burden while remaining easy to administer and interpret. The consistent use of this response format may also facilitate comparisons across studies employing different AI attitude instruments. In contrast to the convergence observed in response formats, substantial variation was evident in both the dimensional structures and item numbers of the reviewed instruments. Although two-dimensional scales were the most frequently reported, AI attitude was operationalized using structures ranging from one to six dimensions. Likewise, scale length varied considerably across studies, indicating that no consensus has yet emerged regarding the optimal level of measurement detail. As demonstrated by the content analysis, this structural diversity likely reflects differences in how AI attitude has been conceptualized rather than merely methodological preferences.

However, the most important contribution of the present review extends beyond the methodological characteristics of AI attitude scales. Although the reviewed studies showed increasing convergence in psychometric practices, they differed substantially in how AI attitude was conceptualized. This distinction is particularly important because methodological similarity does not necessarily imply conceptual similarity. Studies may employ comparable validation procedures, report similar psychometric evidence, and identify analogous factor structures while nevertheless assessing different objects of evaluation, relying on different theoretical perspectives, or representing AI attitude through different dimensions. Consequently, the psychometric quality of an instrument alone is insufficient for understanding what it actually measures. The findings further indicate that conceptualization varies along multiple levels. At the broadest level, some instruments conceptualize attitudes toward AI as a general evaluation of AI as a technology, whereas others focus on attitudes toward specific AI applications within particular professional or educational contexts. Even among instruments sharing the same dimensional structure, substantial conceptual differences remain. For example, three-dimensional instruments do not necessarily represent the same underlying construct, as some are grounded in the classical cognitive–affective–behavioral model of attitude, whereas others organize dimensions around ethical principles, functional motives, or domain-specific characteristics. These findings demonstrate that the number of dimensions alone provides limited information about how AI attitude has actually been conceptualized.

Taken together, the findings suggest that contemporary AI attitude measurement is characterized by bibliographic and procedural commonality without corresponding conceptual uniformity. Studies increasingly draw on common scale families and overlapping cited literatures, and certain methodological practices recur across the corpus, yet substantial disagreement remains regarding what should constitute an attitude toward AI and how that construct should be represented psychometrically. Future progress therefore depends less on continuing to produce new instruments for already represented purposes and more on strengthening the theoretical and cumulative evidence surrounding existing measures. New scale development is most defensible when a clearly specified conceptual or contextual gap cannot be adequately addressed by existing instruments. Otherwise, priority should be given to independent validation, explicit construct definition, stronger assessment of convergent and discriminant validity, test–retest evidence where appropriate, measurement invariance, cross-cultural comparisons, longitudinal and nomological validation, and direct comparisons among competing instruments. Such an approach would help move AI attitude measurement from parallel instrument development toward a more cumulative research program in which theoretical definitions, measurement models, and empirical evidence are more closely aligned.

## Limitations

The findings of this review should be interpreted within the boundaries of its predefined scope. First, the literature search was restricted to the WoS and included only articles published in journals indexed in SCI-EXPANDED, SSCI, A&HCI, or ESCI. This decision provided a consistent bibliographic source base for the systematic mapping and bibliometric analyses, but relevant studies indexed exclusively in other databases or disseminated as conference papers, dissertations, book chapters, preprints, or other forms of gray literature were not represented. Second, the search strategy required AI-related terminology together with “scale” or “instrument” to appear in the title. This approach produced a focused and reproducible corpus in which psychometric measurement was an explicit focus of the publication, but it may have omitted relevant studies whose titles did not contain these terms. For example, Lukić et al. (2023) met the other eligibility criteria and examined attitudes toward AI using a psychometric instrument but was not retrieved by the predefined title-based search. The findings should therefore be understood as representing the predefined WoS-derived corpus rather than the complete universe of AI attitude measurement studies. Third, only English-language publications were included. This restriction may have excluded AI attitude scales developed, adapted, or validated exclusively in other languages. Fourth, the review was deliberately limited to studies published from 2022 onward to examine contemporary developments in AI attitude measurement. Consequently, earlier instrument-development studies were not included in the descriptive and conceptual synthesis conducted within the systematic mapping review. Some foundational publications, including Schepman and Rodway (2020) and Sindermann et al. (2021), nevertheless remained visible in the co-citation analysis because they form part of the cited intellectual foundations of the contemporary literature. Accordingly, the review should not be interpreted as providing a historical account of the full development of AI attitude measurement. Fifth, the search was conducted on June 24, 2026. Consequently, the publication count for 2026 represents only studies indexed up to that date and should not be interpreted as a complete annual total. Additional AI attitude scale studies may have appeared after the search date, meaning that the findings provide a time-bounded representation of an actively developing literature. Finally, the present review characterized the methodological and conceptual features of the included studies but did not conduct a formal instrument-level appraisal of the methodological quality of individual validation studies or the strength of evidence supporting specific measurement properties. The findings therefore do not provide a basis for ranking individual instruments according to psychometric quality or for identifying a single preferred measure. Rather, the review maps how AI attitude measurement has been conceptualized, methodologically approached, and organized within the contemporary literature. A subsequent systematic review of measurement properties using a dedicated appraisal framework could complement the present mapping review by directly comparing the strength and consistency of evidence supporting established instruments.

## Conclusion

This systematic mapping review, integrated with BMA, examined contemporary AI attitude scale research within a predefined WoS-derived corpus. Based on 51 AI attitude studies included in the final analytic sample, published between 2022 and June 2026, the review examined publication and methodological patterns, the intellectual and bibliographic structure of the literature, and the ways in which AI attitude has been conceptualized across existing instruments. The findings indicate substantial recent growth in AI attitude measurement research within the reviewed corpus, accompanied by continued development of new instruments and increasing efforts to adapt and validate established measures across populations and contexts.

The central contribution of the review lies in demonstrating that bibliographic relatedness does not imply conceptual equivalence. Co-citation analysis showed that contemporary AI attitude measurement draws on both the broader technology-acceptance tradition and AI-specific measurement research, while bibliographic coupling revealed several interconnected contemporary research fronts built on overlapping cited knowledge bases. At the same time, the conceptual synthesis identified substantial heterogeneity across instruments in their theoretical foundations, attitude objects, dimensional structures, and substantive content. Instruments bearing the same general label of artificial intelligence attitude, or even sharing similar dimensional structures, may therefore operationalize meaningfully different psychological content.

These findings have direct implications for future measurement research. Rather than selecting an instrument primarily on the basis of its title, popularity, dimensionality, or previous use, researchers should consider whether its conceptual definition, attitude object, dimensional structure, target population, and application context correspond to the purpose of the study. New scale development is most warranted when an existing instrument does not adequately represent a clearly specified conceptual or contextual need. Otherwise, greater cumulative value may be gained by strengthening evidence around established measures through independent validation, measurement invariance testing, cross-cultural comparison, longitudinal and nomological validation, and direct comparisons among alternative instruments. Formal comparative appraisal of measurement-property evidence would further complement the present mapping by clarifying the relative evidential support for established AI attitude scales. Overall, progress in AI attitude measurement will depend not only on the availability of psychometrically evaluated instruments, but also on clearer construct boundaries and stronger alignment among theoretical definitions, measurement models, and intended applications.

## Data Availability

The original contributions presented in the study are included in the article/Supplementary material, further inquiries can be directed to the corresponding author.

## References

[B1] Ahmadi Fatalaki J. Saeedi Z. Sheikhzadeh Marand E. Nasiri Firuz A. R. (2025). Artificial intelligence in education and EFL teachers' attitudes: scale development and validation. Innov. Lang. Learn. Teach. 24, 1–17. doi: 10.1080/17501229.2025.2494823

[B2] Amiri H. Peiravi S. Rezazadeh Shojaee S. S. Rouhparvarzamin M. Nateghi M. N. Etemadi M. H. . (2024). Medical, dental, and nursing students' attitudes and knowledge towards artificial intelligence: a systematic review and meta-analysis. BMC Med. Educ. 24:412. doi: 10.1186/s12909-024-05406-1 38622577 PMC11017500

[B3] Arees Z. Guntín S. Fariña F. Novo M. (2025). Spanish adaptation and validation of the general attitudes towards artificial intelligence scale (GAAIS). European J. Investigat. Health Psychol. Educ. 15:230. doi: 10.3390/ejihpe15110230 41294790 PMC12651838

[B4] Aria M. Cuccurullo C. (2017). Bibliometrix: an R-tool for comprehensive science mapping analysis. J. Informetr. 11, 959–975. doi: 10.1016/j.joi.2017.08.007

[B5] Arslan N. Esin K. Ayyildiz F. (2025). A validity and reliability study of the artificial intelligence attitude scale (AIAS-4) and its relationship with social media addiction and eating behaviors in Turkish adults. BMC Public Health 25:1244. doi: 10.1186/s12889-025-22507-8 PMC11963498 40175951

[B6] Aydin Yildiz T. Çinar Yagci S. Orhan A. (2026). Development and validation of generative artificial intelligence attitude scale for EFL teachers (GenAIAS-EFL teachers form). Interact. Learn. Environ. 34, 3907–3928. doi: 10.1080/10494820.2025.2583196

[B7] Azman N. A. Rasudin N. S. Ismail R. Ghafar N. A. Shohaimi S. Ahmad N. S. . (2025). Validation of the Bahasa Malaysia version of the general attitudes towards artificial intelligence scale among academicians. Malaysian J. Med. Sci. 32, 103–114. doi: 10.21315/mjms-05-2025-336 PMC13132139 42077245

[B8] Bagočiunaite R. Endriulaitiene A. (2026). Understanding employee attitudes toward artificial intelligence in the workplace: a systematic review of attitude definitions and measurements. Business: Theory and Pract. 27, 168–178. doi: 10.3846/btp.2026.25127

[B9] Beghetto R. A. Ross W. Karwowski M. Glăveanu V. P. (2025). Partnering with AI for instrument development: possibilities and pitfalls. New Ideas Psychol. 76:101121. doi: 10.1016/j.newideapsych.2024.101121

[B10] Bilge Y. Emiral E. Delal I. (2025). Attitudes towards artificial intelligence (AI) in psychotherapy: artificial intelligence in psychotherapy scale (AIPS). Clin. Psychol. 29, 194–204. doi: 10.1080/13284207.2025.2515074

[B11] Boduroglu E. Yigiter M. S. (2026). Artificial intelligence scoring attitudes: scale development and validation. Educ. Inform. Technol. 31, 701–726. doi: 10.1007/s10639-025-13836-7

[B12] Breckler S. J. (1984). Empirical validation of affect, behavior, and cognition as distinct components of attitude. J. Pers. Soc. Psychol. 47, 1191–1205. doi: 10.1037/0022-3514.47.6.1191 6527214

[B13] Chen T.-J. (2023). ChatGPT and other artificial intelligence applications speed up scientific writing. J. Chinese Med. Assoc. 86, 351–353. doi: 10.1097/JCMA.0000000000000900 PMC12755446 36791246

[B14] Cheng Y.-C. Lu C.-Y. Chen K.-J. Lin C.-T. (2025). Development and validation of an attitude scale toward generative AI for university students. Int. J. Eng. Educ. 41, 1556–1568.

[B15] Cicero L. Russo A. Di Stefano G. Zammitti A. (2025). The general attitudes towards artificial intelligence scale (GAAIS): validation and psychometric properties analysis in the Italian context. BMC Psychol. 13:641. doi: 10.1186/s40359-025-02935-2 PMC12211962 40597372

[B16] Cipriani E. Menicucci D. Greco A. Grassini S. (2026). Emotional responses to AI use: development of the SAG-AI scale for shame and guilt. Int. J. Human–Comp. Interact. 84, 1–20. doi: 10.1080/10447318.2026.2648803

[B17] Davis F. D. (1989). Perceived usefulness, perceived ease of use, and user acceptance of information technology. MIS Quarter. 13, 319–340. doi: 10.2307/249008

[B18] Derinalp P. Ozyurt M. (2025). Adaptation of the student attitudes toward artificial intelligence scale to the Turkish context: validity and reliability study. Int. J. Human–Comp. Interact. 41, 4653–4667. doi: 10.1080/10447318.2024.2352921

[B19] Durak G. Öncü S. Çankaya S. Çigdem H. (2026). Development and validation of the scale for attitudes towards generative AI (SAGAI). Eur. J. Educ. 61:e70415. doi: 10.1111/ejed.70415

[B20] Eagly A. H. Chaiken S. (1993). The Psychology of Attitudes. New York, NY: Harcourt Brace Jovanovich College Publishers.

[B21] Ellegaard O. Wallin J. A. (2015). The bibliometric analysis of scholarly production: how great is the impact? Scientometrics 105, 1809–1831. doi: 10.1007/s11192-015-1645-z 26594073 PMC4643120

[B22] Facca L. Béna J. (2026). Internal validation of the French attitudes toward AI in defense (AAID) scale: evidence from an open dataset. Acta Psychol. 263:106366. doi: 10.1016/j.actpsy.2026.106366 41643336

[B23] Grassini S. (2023). Development and validation of the AI attitude scale (AIAS-4): a brief measure of general attitude toward artificial intelligence. Front. Psychol. 14:1191628. doi: 10.3389/fpsyg.2023.1191628 PMC10406504 37554139

[B24] Hadlington L. Binder J. Gardner S. Karanika-Murray M. Knight S. (2023). The use of artificial intelligence in a military context: development of the attitudes toward AI in defense (AAID) scale. Front. Psychol. 14:1164810. doi: 10.3389/fpsyg.2023.1164810 PMC10196355 37213385

[B25] Hering M. Neef N. E. Zabel S. Otto S. (2026). General artificial intelligence attitude scale (GAIA-15)–How to measure attitude toward an ever-changing object. Comput. Hum. Behav. Rep. 22:101096. doi: 10.1016/j.chbr.2026.101096

[B26] Hu R. Ma Y.-F. Wang C.-Y. Li X.-Y. Huang H.-P. (2025). Validity and reliability of the Chinese translation of the attitude scale towards the use of artificial intelligence technologies in nursing (ASUAITIN)- a cross-sectional study. BMC Nurs. 24:782. doi: 10.1186/s12912-025-03322-3 PMC12211220 40597054

[B27] Huang Y. Jiang S. Gong Z. (2025). Validity and reliability of the Chinese version of general attitudes towards artificial intelligence scale. Int. J. Human–Comp. Interact. 41, 12884–12894. doi: 10.1080/10447318.2025.2465868

[B28] Hutzschenreuter T. Lämmermann T. Sake A. Ludwig H. (2025). Successfully mitigating AI management risks to scale AI globally. MIS Quarter. Executive 24:3. doi: 10.17705/2msqe.00117

[B29] Juranek J. F. Pochwatko G. (2026). The Polish adaptation of the artificial intelligence attitude scale: AIAS-4-PL. Curr. Psychol. 45:410. doi: 10.1007/s12144-025-08930-5

[B30] Kantor J. Morrison M. Ko J. Semenov Y. R. Fortgang R. G. (2026). Development and validation of the Oxford attitudes to artificial intelligence in medicine scale. JAAD Int. 26, 113–121. doi: 10.1016/j.jdin.2026.04.005 42179892 PMC13196147

[B31] Karaarslan D. Ergin E. (2026). Attitudes toward artificial intelligence in healthcare: pediatrics scale validity and reliability study of the Turkish version. Int. J. Human–Comp. Interact. 42, 3706–3718. doi: 10.1080/10447318.2025.2534063

[B32] Kaya F. Aydin F. Schepman A. Rodway P. Yetişensoy O. Demir Kaya M. . (2024). The roles of personality traits, AI anxiety, and demographic factors in attitudes toward artificial intelligence. Int. J. Human–Comp. Interact. 40, 497–514. doi: 10.1080/10447318.2022.2151730

[B33] Khorasanizadeh M. Shahbandi A. Maroufi S. F. Taghipour P. Sayedsalehi S. Zeinaddini-Meymand A. . (2024). Trends of neurosurgical publications in high-impact medical journals: a bibliometric study. World Neurosurg. 190, 443–450. doi: 10.1016/j.wneu.2024.07.205 39111658

[B34] Köse N. Simşek E. Demir M. C. (2025). Adaptation of artificial intelligence attitude scale (AIAS-4) into Turkish: a validity and reliability study. Curr. Psychol. 44, 8096–8105. doi: 10.1007/s12144-025-07418-6

[B35] Li A. Wang S. (2026). Functional attitudes towards artificial intelligence among university students: development of a scale and influencing factors. Higher Educ. 91, 1815–1837. doi: 10.1007/s10734-025-01497-x

[B36] Lim W. M. Gunasekara A. Pallant J. L. Pallant J. I. Pechenkina E. (2023). Generative AI and the future of education: ragnarök or reformation? A paradoxical perspective from management educators. Int. J. Manag. Educ. 21:100790. doi: 10.1016/j.ijme.2023.100790

[B37] Lukić A. Kudelić N. Antičević V. Lazić-Mosler E. Glunčić V. Hren D. . (2023). First-year nursing students' attitudes towards artificial intelligence: cross-sectional multi-center study. Nurse Educ. Pract. 71:103735. doi: 10.1016/j.nepr.2023.103735 37541081

[B38] Marengo A. Karaoglan-Yilmaz F. G. Yilmaz R. Ceylan M. (2025). Development and validation of generative artificial intelligence attitude scale for students. Front. Comp. Sci. 7:1528455. doi: 10.3389/fcomp.2025.1528455

[B39] Martins J. Gonçalves R. Branco F. (2024). A bibliometric analysis and visualization of e-learning adoption using VOSviewer. Univers. Access Inf. Soc. 23, 1177–1191. doi: 10.1007/s10209-022-00953-0 36530863 PMC9734988

[B40] Mirsch M. Knoth N. Schultz B. Bata K. Schleiss J. Carmen L.-S. . (2026). Measuring attitudes toward responsible AI in engineering–development and validation of the RAISE scale. European J. Eng. Educ. 27, 1–23. doi: 10.1080/03043797.2026.2620482

[B41] Morales-García W. C. Sairitupa-Sanchez L. Z. Morales-García S. B. Morales-García M. (2024). Adaptation and psychometric properties of an attitude toward artificial intelligence scale (AIAS-4) among Peruvian nurses. Behav. Sci. 14:437. doi: 10.3390/bs14060437 38920769 PMC11200830

[B42] Novotny M. Weber W. Kern C. Kreuter F. (2026). Measuring public opinion towards artificial intelligence: development and validation of a general AI attitude short scale. AI Soc. 41, 4227–4260. doi: 10.1007/s00146-025-02478-5

[B43] OECD (2024). Assessing Potential Future Artificial Intelligence Risks, Benefits And Policy Imperatives (OECD Artificial Intelligence Papers No. 27). Paris: OECD Publishing.

[B44] Olpak Y. Z. (2022). Community of inquiry framework: research trends between 2000-2020. Online Learn. 26, 350–368. doi: 10.24059/olj.v26i2.2737

[B45] Olpak Y. Z. Yilmaz R. (2022). Review of trends in peer instruction: bibliometric mapping analysis and systematic review. J. Learn. Teach. Digital Age 7, 42–50. doi: 10.53850/joltida.978824

[B46] Orhan A. Aydin Yildiz T. Çinar Yagci S. (2026). Assessing EFL learners' attitudes on generative artificial intelligence: development and validation of generative artificial intelligence attitude scale for EFL learners (GenAIAS). J. Res. Technol. Educ. 58, 494–514. doi: 10.1080/15391523.2024.2437744

[B47] O'Shaughnessy M. R. Schiff D. S. Varshney L. R. Rozell C. J. Davenport M. A. (2023). What governs attitudes toward artificial intelligence adoption and governance? Sci. Public Policy 50, 161–176. doi: 10.1093/scipol/scac056

[B48] Öztürk Yildirim T. Karaman M. (2025). Development and psychometric evaluation of the artificial intelligence attitude scale for nurses. BMC Nurs. 24:441. doi: 10.1186/s12912-025-03098-6 40264200 PMC12013020

[B49] Page M. J. McKenzie J. E. Bossuyt P. M. Boutron I. Hoffmann T. C. Mulrow C. D. . (2021). The PRISMA 2020 statement: an updated guideline for reporting systematic reviews. BMJ, 372:71. doi: 10.1136/bmj.n71 33782057 PMC8005924

[B50] Park J. Woo S. E. Kim J. (2024). Attitudes towards artificial intelligence at work: scale development and validation. J. Occup. Organ. Psychol. 97, 920–951. doi: 10.1111/joop.12502

[B51] Rabkin S. W. (2026). Artificial intelligence in the assessment of heart rate variability as an instrument to understand the connection between psychologic and psychiatric conditions and the heart. Bioengineering 13:554. doi: 10.3390/bioengineering13050554 42194311 PMC13203877

[B52] Roche V. Daynac J. Hesni S. Geymond U. Ginzburg N. Philippon J. . (2026). Global-scale AI-powered prediction of hydrogen seeps. Mar. Pet. Geol. 183:107617. doi: 10.1016/j.marpetgeo.2025.107617

[B53] Rózsa S. Bandi S. Hartung I. Török I. A. Varga J. É. Somlai E. H. . (2025). General attitudes towards artificial intelligence scale (GAAIS): hungarian adaptation and links to personality traits. Front. Psychol. 16:1703750. doi: 10.3389/fpsyg.2025.1703750 41323901 PMC12662174

[B54] Sacco F. Manzi F. Di Dio C. Schepman A. Rodway P. Massaro D. . (2025). Validation of the general attitudes towards artificial intelligence scale in the Italian context and the role of trust. Comput. Hum. Behav. Rep. 19:100751. doi: 10.1016/j.chbr.2025.100751

[B55] Sarun H. Rotana S. Chhunla C. (2026). The role and significance of artificial intelligence in transforming modern society: opportunities, challenges, and future directions. J. Agricult. Environ. 3, 133–141. doi: 10.5281/zenodo.18258452

[B56] Satici S. A. Okur S. Yilmaz F. B. Grassini S. (2025). Psychometric properties and Turkish adaptation of the artificial intelligence attitude scale (AIAS-4): evidence for construct validity. BMC Psychol. 13:297. doi: 10.1186/s40359-025-02505-6 PMC11934756 40128815

[B57] Schepman A. Rodway P. (2020). Initial validation of the general attitudes towards artificial intelligence scale. Comp. Human Behav. Rep. 1:100014. doi: 10.1016/j.chbr.2020.100014 PMC7231759 34235291

[B58] Schepman A. Rodway P. (2023). The general attitudes towards artificial intelligence scale (GAAIS): confirmatory validation and associations with personality, corporate distrust, and general trust. Int. J. Human–Comp. Interact. 39, 2724–2741. doi: 10.1080/10447318.2022.2085400

[B59] Schepman A. Rodway P. (2026). Validation of the short general attitudes towards artificial intelligence scale: the short GAAIS-10. Int. J. Human–Comp. Interact. 42, 1–17. doi: 10.1080/10447318.2025.2610446

[B60] Sindermann C. Sha P. Zhou M. Wernicke J. Schmitt H. S. Li M. . (2021). Assessing the attitude towards artificial intelligence: introduction of a short measure in German, Chinese, and English language. KI-Künstliche Intelligenz 35, 109–118. doi: 10.1007/s13218-020-00689-0

[B61] Stein J.-P. Messingschlager T. Gnambs T. Hutmacher F. Appel M. (2024). Attitudes towards AI: measurement and associations with personality. Sci. Rep. 14:2909. doi: 10.1038/s41598-024-53335-2 PMC10844202 38316898

[B62] Suárez Á. Specht M. Prinsen F. Kalz M. Ternier S. (2018). A review of the types of mobile activities in mobile inquiry-based learning. Comp. Educ. 118, 38–55. doi: 10.1016/j.compedu.2017.11.004

[B63] Suh W. Ahn S. (2022). Development and validation of a scale measuring student attitudes toward artificial intelligence. Sage Open 12, 1–12. doi: 10.1177/21582440221100463

[B64] Sultana A. Latheef N. A. Siby N. Ahmad Z. (2025). Exploring students' attitudes toward artificial intelligence (AI): psychometric validation of AI-attitude scale. Sage Open 15:21582440251378375. doi: 10.1177/21582440251378375

[B65] Svetlanova S. Iskakova B. Makhanbetkulova D. Blazeviciene A. Nazarova L. Aitambayeva N. . (2026). Assessing nurses' attitudes toward artificial intelligence in Kazakhstan: psychometric validation of a nine-item scale. Front. Digit. Health 8:1756338. doi: 10.3389/fdgth.2026.1756338 41993039 PMC13081779

[B66] Talik W. Talik E. B. Grassini S. (2025). Measurement invariance of the artificial intelligence attitude scale (AIAS-4): cross-cultural studies in Poland, the USA, and the UK. Curr. Psychol. 44, 15758–15766. doi: 10.1007/s12144-025-08348-z

[B67] Tsang T. Yin C. H. (2026). Enhance solar panel efficiency with a scale-down model and using artificial intelligence (AI) model. Int. J. Software Eng. Knowledge Eng. 36, 261–276. doi: 10.1142/S0218194025500755

[B68] Turan M. Cengiz Z. (2025). Nursing students' general attitudes towards artificial intelligence scale (NGAAIS): a Turkish validity and reliability study. Nurse Educ. Pract. 88:104574. doi: 10.1016/j.nepr.2025.104574 41045905

[B69] Turgut D. Kunuroglu F. (2025). Adaptation of the student attitudes toward artificial intelligence scale (SATAI) to the Turkish context: a sample of emerging adults. International J. Human–Comp. Interact. 41, 13505–13515. doi: 10.1080/10447318.2025.2474474

[B70] Wallin J. A. (2005). Bibliometric methods: pitfalls and possibilities. Basic Clin. Pharmacol. Toxicol. 97, 261–275. doi: 10.1111/j.1742-7843.2005.pto_139.x 16236137

[B71] Wu F. Dang Y. Li M. (2025). A systematic review of responses, attitudes, and utilization behaviors on generative AI for teaching and learning in higher education. Behav. Sci. 15:467. doi: 10.3390/bs15040467 40282088 PMC12023922

[B72] Yamaguchi Y. Hashimoto C. Saito N. (2025). GAAIS-J: translation and validation of the Japanese version of the general attitudes toward artificial intelligence scale. Behav. Sci. 15:1668. doi: 10.3390/bs15121668 41464011 PMC12730081

[B73] Yilmaz D. Uzelli D. Dikmen Y. (2025). Psychometrics of the attitude scale towards the use of artificial intelligence technologies in nursing. BMC Nurs. 24:151. doi: 10.1186/s12912-025-02732-7 39930420 PMC11809082

[B74] Zawacki-Richter O. Latchem C. (2018). Exploring four decades of research in Computers and Education. Comp. Educ. 122, 136–152. doi: 10.1016/j.compedu.2018.04.001

